# PPIA, HPRT1, and YWHAZ Genes Are Suitable for Normalization of mRNA Expression in Long-Term Expanded Human Mesenchymal Stem Cells

**DOI:** 10.1155/2019/3093545

**Published:** 2019-05-21

**Authors:** Ryoung-Hoon Jeon, Won-Jae Lee, Young-Bum Son, Dinesh Bharti, Sharath Belame Shivakumar, Sung-Lim Lee, Gyu-Jin Rho

**Affiliations:** ^1^College of Veterinary Medicine, Gyeongsang National University, Jinju 52828, Republic of Korea; ^2^College of Veterinary Medicine, Kyungpook National University, Daegu 41566, Republic of Korea

## Abstract

Long-term expansion of mesenchymal stem cells (MSCs) under defined culture conditions is necessary in human stem cell therapy. However, it alters the characteristics of MSCs. Since quantitative real time polymerase chain reaction (qRT-PCR) is widely used as one of the key analytical methods for comparative characterization, the validation of reference genes (RGs) for normalization under each experimental condition is important to achieve reliable qRT-PCR results. Therefore, the most stable RGs for long-term expanded bone marrow- and umbilical cord blood-derived MSCs (BM-MSCs and UCB-MSCs) under defined culture conditions for up to 20 passages were evaluated. The more apparent alterations in characteristics such as differentiation capacity, proliferation, senescence, and the expression of RGs were noted in BM-MSCs than UCB-MSCs during long-term expansion. The RG validation programs (GeNorm and NormFinder) suggested that PPIA, HPRT1, and YWHAZ were suitable for normalization in qRT-PCR regardless of MSC types and long-term culture expansion, and the traditional RGs (ACTB and GAPDH) were less stable in long-term expanded MSCs. In addition, the use of these RGs for normalization of OCT4 expression in long-term expanded BM-MSCs showed that a less stable RG (GAPDH) showed contrasting data compared to other RGs. Therefore, the use of RGs such as PPIA, HPRT1, and YWHAZ for normalization in qRT-PCR experiments is highly recommended for long-term expanded MSCs to generate accurate and reliable data.

## 1. Introduction 

Since gene expression studies have enabled us to understand the gene-regulatory network of cellular mechanisms, the quantitative analysis of gene expression is essential in cellular biology [1]. Among commonly used techniques, the quantitative real time polymerase chain reaction (qRT-PCR) has been the most extensively employed technique to monitor gene expression owing to its sensitivity, specificity, reproducibility, large dynamic range, and high degree of automation [2–4]. In qRT-PCR, the normalization of genes of interest (GOI) against a reference gene (RG) is imperative to investigate the relative quantification of gene expression, since it is believed that RGs are stably and constantly expressed in cells regardless of experimental conditions [4–6]. Unfortunately, there are no reports on using a single universal RG having constant expression. It has been uncovered that the expression of commonly used RGs even varies depending on cell type, cell source, and experimental conditions. Furthermore, RG expression is also affected by the quality of obtained RNA, cDNA synthesis conditions, type of detection chemistry, and inhibitory contaminants [6–10]. Since the selection of inadequate and unstable RGs for normalization of GOI may lead to the generation of false data or contradictable results, the validation of most stable RGs from the pool of widely used RGs under each experimental setup is prerequisite for obtaining accurate and reliable results by qRT-PCR [5, 11].

Mesenchymal stem cells (MSCs) are being highlighted as promising candidates for stem cell therapy and have made a significant impact in the field of regenerative medicine due to their self-renewal property, wide accessibility, multilineage differentiation potential, and immunomodulation properties [12]. In this context, bone marrow-derived MSCs (BM-MSCs) have been extensively studied in the past decades. However, the isolation of BM-MSCs requires invasive procedures and these cells are associated with certain limitations such as age-dependent decline in their proliferation and differentiation ability [13]. Therefore, the umbilical cord blood-derived MSCs (UCB-MSCs) serves as potential alternatives to BM-MSCs because UCB specimens containing several primary MSC populations are being cryopreserved throughout the world [14, 15].

For the clinical application of MSCs, several obstacles have to be overcome which occur during cell culture. The isolated primary MSCs have to be expanded extensively* in vitro* to achieve a sufficient quantity for therapeutic purposes due to their occurrence in low population in many human tissues. However, the* in vitro*-expanded MSCs are not immortal and consequently undergo cellular alterations such as loss of proliferative ability and differentiation potential, replicative senescence, and change in expression of genes including RGs [16, 17]. In addition, several efforts have been made to replace animal-derived supplements like fetal bovine serum (FBS) with defined culture conditions such as serum- and xeno-free media to prevent the possible adverse effects such as severe immune reactions, viral and bacterial infections, and zoonoses [18, 19]. Since* in vitro* environments (*in vitro* long-term expansion, serum- and xeno-free media) may also influence the cellular characteristics, the cellular alterations in long-term expanded MSCs under defined culture conditions should be studied further for safety, reliability, and efficacy prior to the clinical applications. Recently, it has been highly emphasized that the selection of appropriate RGs under each experimental condition for MSC research is an essential step to obtain reliable results before performing qRT-PCR [4–6, 20]. However, to the best of our knowledge, the selection of suitable RGs for long-term expanded MSCs under defined culture conditions is not well studied. Therefore, the present study aims to investigate the most stable RGs to provide an accurate evaluation of relative mRNA fold change of GOI in long-term expanded human MSCs (BM-MSCs and UCB-MSCs) under defined culture conditions. Ten commonly used RGs (18S, B2M, EEF1A1, GAPDH, HPRT1, PPIA, RPLP0, TBP, ACTB, and YWHAZ) were studied and compared to determine the appropriate RGs for long-term expanded BM-MSCs and UCB-MSCs under defined culture conditions through widely used RG validation programs: GeNorm and NormFinder.

## 2. Materials and Methods

### 2.1. Chemicals and Ethics

All chemicals and media were purchased from Thermo Fisher Scientific (Waltham, MA, USA), unless otherwise specified. All volunteers for specimen donation were given written informed consent, and the Ethics Committee of the Gyeongsang National University Hospital (GNUH-2012-09-004) approved the present study.

### 2.2. Characterization of Long-Term Expanded BM-MSCs and UCB-MSCs

BM aspirates or UCB were obtained during surgery or parturition to isolate BM-MSCs (n = 5, male) or UCB-MSCs (n = 5, male), respectively. The MSCs were then cultured in defined culture media (StemPro MSC SFM Xenofree medium supplemented with L-alanyl-L-glutamine, 100 unit/ml Penicillin and 100 *μ*g/ml Streptomycin) and detached with TrypLE during subculture for long-term expansion. In brief, BM aspirates and UCBs were diluted with Dulbecco's phosphate buffered saline (DPBS; 1:1 (v/v)) and layered on Ficoll (density 1.077 g/cm^3^, GE Healthcare, IL, USA). After centrifugation (400 ×g, 30 min) at 4°C, the mononuclear cell (MNC) fraction was harvested, plated onto a 6-well plate at 1 × 10^5^ cells/cm^2^ in defined culture media, and cultured at 37°C in a humidified atmosphere of 5%  CO_2_ as previously reported [13]. Once the adherent primary MSC populations reached 80% confluence, they were harvested with TrypLE and passaged until 20 passages with defined culture media. The expanded BM-MSCs were categorized as early passage (E-BM-MSCs), mid passage (M-BM-MSCs), or late passage BM-MSCs (L-BM-MSCs) at passage 2-4, 10-12, or 18-20, respectively. Similarly, UCB-MSCs were also categorized as early passage (E-UCB-MSCs), midpassage (M-UCB-MSCs), and late passage UCB-MSCs (L-UCB-MSCs) at passage 2-4, 10-12, or 18-20, respectively. The MSCs in all groups were analyzed for their expression of MSC-specific surface markers (CD44, CD105, and vimentin) and the absence of the expression of hematopoietic stem cell markers (CD34 and CD45). In brief, MSCs at 80% confluence were harvested, and fixed in 4% paraformaldehyde for overnight. The cells were then washed twice with PBS and incubated with fluorescein isothiocyanate (FITC)-conjugated mouse anti-human CD34 (1:200; BD Biosciences, NJ, USA), mouse anti-human CD44 (1:200; BD Biosciences), mouse anti-human CD45 (1:200; BD Biosciences), mouse anti-human CD105 (1:200; BD Biosciences), and Alexa Fluor® 488 mouse anti-human vimentin (1:200; BD Biosciences). A total of 1 × 10^4^ FITC-labeled MSCs were acquired and analyzed using BD FACS Calibur (BD Biosciences). The differentiation ability of all MSCs toward mesenchymal lineages was evaluated following previous protocols [12, 21]. Briefly, osteogenic differentiation was induced for 3 weeks with Dulbecco's Modified Eagle Medium (DMEM) supplemented with 10% FBS, 200 mM ascorbic acid, 10 mM *β*-glycerophosphate, and 0.1 mM dexamethasone, and osteogenesis was confirmed by observing the calcium deposits after staining with 5% sliver nitrate solution (Von Kossa staining). Chondrogenesis was induced by using STEMPRO chondrogenesis differentiation kit. Briefly, 1 × 10^6^ MSCs were suspended in 15 mL tube and were centrifuged at 450 ×g for 5 min. The pellets were cultured for 3 weeks in STEMPRO chondrogenesis media. The pellets were then used for preparing paraffin-embedded sections on glass slides. The sections were stained with 1% alcian blue solution for proteoglycan detection and were counterstained with 0.1% nuclear fast red solution. The MSCs were cultured for 3 weeks in DMEM supplemented with 10% FBS, 100 mM indomethacin, 10 mM insulin, and 1 mM dexamethasone for the adipogenesis. The differentiation into adipocyte lineage was confirmed by evaluating the accumulation of intracellular lipid vacuoles after staining with 0.5% Oil red O solution.

### 2.3. Proliferative Potential and *β*-Galactosidase Activity in Long-Term Expanded BM-MSCs and UCB-MSCs

The Vybrant® MTT [3-(4,5-dimethylthiazol-2-yl)-2,5-diphenyltetrazolium bromide] Cell Proliferation Assay was used for the comparative evaluation of proliferative potential of MSCs. Briefly, 1 × 10^3^ MSCs were cultured in 96 well plate and every other day cells were incubated with 20 *μ*l MTT solution (5 mg/ml) for 4 hr. After incubation, the supernatant was removed and 50 *μ*l dimethyl sulfoxide (DMSO) was added. The product formed was then transferred to 96 well plate and the absorbance was measured at 540 nm using microplate reader (VersaMax™, Molecular Devices, CA, USA). The senescence-associated *β*-galactosidase activity (SA-*β*-gal) was measured using *β*-galactosidase Assay Kit following manufacturer's protocol. Briefly, cells at 70-80% confluence were harvested by treating with TrypLE and washed twice with DPBS, and a total of 5 × 10^5^ cells were treated with 200 *μ*l M-PER reagent and then gently agitated for 10 min. After being centrifuged at 27,000 ×g for 15 min to remove the debris of cell lysis, 50 *μ*l of the supernatant was transferred into a 96-well microplate. The cell extract was incubated at 37°C for 30 min with 50 *μ*l of *β*-galactosidase reagent and the absorbance was measured at 405 nm using microplate reader (VersaMax™, Molecular Devices, CA, USA).

### 2.4. Candidate Reference Genes and Primer Sequences

Ten RGs were chosen based on different intracellular functional activity and in accordance with the previous reports [4–6]. The primers for each RG were designed by Primer 3 Plus software (considering 60°C annealing temperature) and were analyzed for the formation of hairpins, homodimers, and heterodimers using OligoAnalyzer 3.1 software. In order to check the PCR efficiencies, a standard curve of each primer was made from the cycle threshold (Ct) values by a four-fold serial dilution of cDNA (1:10, 1:100, 1:1,000 and 1:10,000) of E-BM-MSCs following the previous protocols [6, 22]. Standard curve parameters such as slope (M), intercept (B), PCR efficiencies (E = 10^-1/slope^-1 × 100), and correlation (R^2^) were calculated using RotorGene Q Series Software (Qiagen, Hilden, Germany) or Excel (Microsoft, WA, USA). The full name, gene symbol, nucleotide sequences, amplicon sizes, accession number, and PCR efficiencies of RG primers are described in Table 1.

### 2.5. RNA Extraction, cDNA Synthesis, and qRT-PCR

The total RNA was extracted from MSCs using RNeasy Mini Kit (Qiagen) in accordance with the manufacturer's instructions with residual genomic DNA removal step by RNase-free DNase treatment (Qiagen). The concentration and purity of RNA sample were measured by assessing A260/A280 ratio using spectrophotometer (NanoDrop 1000, Thermo Fisher Scientific). First-strand cDNA was synthesized from 1 *μ*g of total RNA extract using Omniscript Reverse Transcription Kit (Qiagen) at 37°C for 1 h. For PCR amplification, the reaction mixture containing Rotor-Gene 2X SYBR Green mix (Qiagen), 2 *μ*l cDNA, and 400 nM of each forward and reverse primers was run in a Rotor Gene Q qRT-PCR machine (Qiagen) following the program of pre-denaturation at 95°C for 10 min, 45 cycles at 95°C for 10 s, 60°C for 6 s, and 72°C for 4 s, and a melting curve from 60°C to 95°C by 1°C/s and cooling at 40°C for 30 s. Rotor-Gene Q Series Software (Qiagen) was used for the analysis of amplification curves, melting curves, and cycle threshold values (Ct values). All amplicons from qRT-PCR were checked by gel electrophoresis to verify their expected product size and confirmed the absence of nonspecific amplification.

### 2.6. Analysis of Stable Reference Gene Expression

The experimental groups for analyzing stable RGs throughout long-term culture expansion of MSCs under defined culture conditions were allocated into BM-MSCs (Group 1), UCB-MSCs, (Group 2), and both BM-MSCs and UCB-MSCs (Group 3). The Ct values of each RG were assessed via commonly used RG validation programs: geNorm and NormFinder. The geNorm program measures the gene expression stability (M value), where a gene calculated as the highest M value is excluded from the pool of tested genes as the least stable RG; then the new M values are continuously generated until the last two genes with the lowest M values are left as the most stable RGs. In addition, the GeNorm suggests the normalization factors (NF) for an optimal number of RGs (NF_opt_) with pairwise variation (Vn/n+1) estimated by continuous calculation depending on adding other genes sequentially from 2 genes with the lowest M value [23]. In the present study, correlations between NF_opt_ and NF for three most stable reference genes (NF_3_) were analyzed by Pearson's correlation using PASW Statistics 18 (SPSS inc., NY, USA) to reduce excessive number of RGs during normalization. Using NormFinder the stability and standard errors of the transcriptional variation of the RGs were calculated via analysis of variance (ANOVA)-based model to evaluate both the single most stable RG showing the lowest value and the best combination of two RGs [24].

### 2.7. The Use of Different RGs in the Normalization of GOI

The effect of stability of RGs in the normalization of GOI was evaluated by using different RGs including the most stable RGs in the present study, and the traditional RGs, which are verified as less stable to calculate the relative gene expression of OCT4 in E-BM-MSCs, M-BM-MSCs, and L-BM-MSCs (Table 1). The qRT-PCR for evaluating OCT4 expression was carried out as described above. The Ct values of OCT4 were then normalized to five RGs (ACTB, GAPDH, HPRT1, PPIA, and YWHAZ) using RotorGene Q Series Software (Qiagen).

### 2.8. Statistical Analysis

The one-way ANOVA with Tukey's post hoc test was performed to determine the significant difference among the groups using PASW Statistics 18 (SPSS Inc.). Differences were considered significant when* P *< 0.05 and all data were represented as mean ± SD.

## 3. Results

### 3.1. Comparative Characterization of Long-Term Expanded BM-MSCs and UCB-MSCs

**Figure 1:**
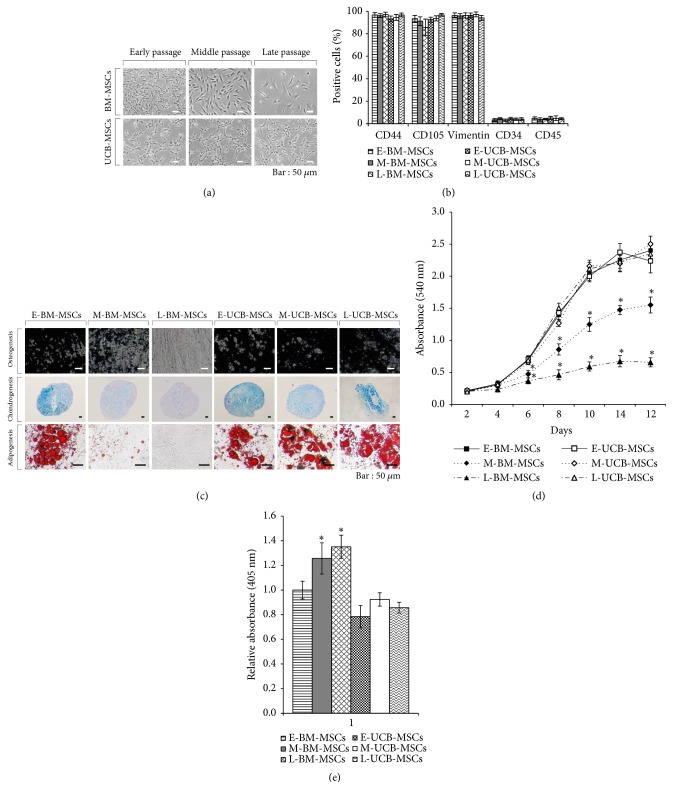
Characterization of UCB-MSCs and BM-MSCs in early passage, and alteration of their features in middle and late passages under defined culture conditions. (a) Morphology of long-term expanded BM-MSCs and UCB-MSCs at early, middle and late passages. Scale bar = 50 *μ*m. (b) Cell surface marker analysis of MSC-positive markers (CD44, CD105, and Vimentin) and negative markers (CD34 and CD45) in long-term expanded BM-MSCs and UCB-MSCs. (c) Lineage-specific differentiation of long-term expanded BM-MSCs and UCB-MSCs. The calcium-rich mineral deposits by osteogenic induction or proteoglycan synthesis by chondrogenic induction or intracellular accumulations of lipid droplets by adipogenic induction was identified by staining with 5% sliver nitrate or alcian blue or Oil-Red O, respectively. Scale bar = 50 *μ*m. (d)* In vitro* proliferation capability of long-term expanded BM-MSCs and UCB-MSCs. Absorbance of MTT metabolite (formazan) was measured at 540 nm every other day from day 2 to day 14. The data were presented as mean ± SD. *∗P *< 0.05 vs E-BM-MSCs. (e) Senescence-associated *β*-galactosidase (SA-*β*-gal) activity in long-term expanded BM-MSCs and UCB-MSCs. Absorbance was measured at 405 nm. The data were presented as mean ± SD, *∗P *< 0.05 vs E-BM-MSCs. E-/M-/L-BM-MSCs, bone marrow-derived mesenchymal stem cells at early passage (passage 2-4)/middle passage (passage 10-12)/late passage (passage 18-20); E-/M-/L-UCB-MSCs, umbilical cord blood mesenchymal stem cell at early passage (passage 2-4)/middle passage (passage 10-12)/late passage (passage 18-20).

The BM-MSCs showed a gradual change in their fibroblastic morphology upon culture expansion; however, UCB-MSCs maintained their fibroblastic morphology until L-UCB-MSCs under defined culture conditions (Figure 1(a)). Both E-BM-MSCs and E-UCB-MSCs were strongly positive for MSC-specific surface markers expression such as CD44, CD105, and vimentin while negative for hematopoietic stem cell markers (CD34 and CD45), and the expression patterns were maintained throughout the long-term culture expansion period. The expression of CD105 in L-BM-MSCs was reduced although not significant (*P* > 0.05) (Figure 1(b) & Supplementary Figures 1 and 2). Further, UCB-MSCs showed differentiation capacity towards mesenchymal lineages such as osteoblasts, chondrocytes, and adipocytes from E-UCB-MSCs to L-UCB-MSCs, whereas, BM-MSCs lost their differentiation potential during long-term expansion as observed in M-BM-MSCs and L-BM-MSCs (Figure 1(c)). Similar to differentiation capacity, proliferative ability and SA-*β*-gal in UCB-MSCs were not affected by long-term expansion when compared to that in long-term expanded BM-MSCs (M-BM-MSCs and L-BM-MSCs), which displayed weaker proliferative ability and increased SA-*β*-gal activity than E-BM-MSCs (Figures 1(d) and 1(e)). Overall, both BM-MSCs and UCB-MSCs maintained their stemness during early passage, and that the long-term expansion of BM-MSCs to later passages under defined culture conditions resulted in alteration of their characteristics and loss of stemness.

### 3.2. Evaluation of Primer Specificity, Amplicon Size, and Ct Values of Selected RGs

**Figure 2:**
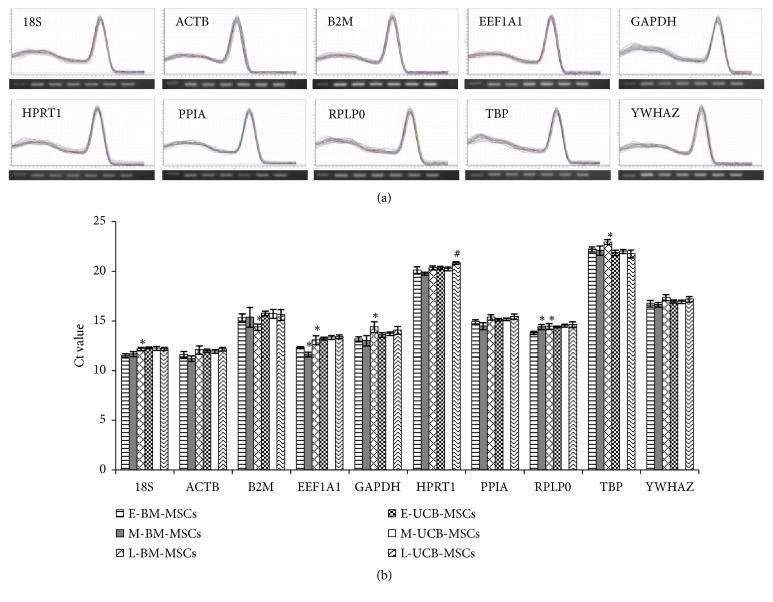
Examination of primer specificity, amplicon size and Ct values of selected reference genes. (a) Melting curves and agarose gel images of PCR amplification products. Specificity of primers and amplicon lengths of 10 selected reference genes (RGs) were analyzed by melting curves and agarose gel electrophoresis (bottom of each reference gene melting curve). Lanes in agarose gel images, which were displayed from the left to the right, E-BM-MSCs, M-BM-MSCs, L-BM-MSCs, E-UCB-MSCs, M-UCB-MSCs, L-UCB-MSCs, and nontemplate control. (b) The Ct values of 10 selected RGs in long-term expanded BM-MSCs and UCB-MSCs. The data were presented as mean ± SD. *∗P* < 0.05 vs E-BM-MSCs and ^#^*P* < 0.05 vs E-UCB-MSCs.

The standard curves were generated to check primer efficiency, which resulted in 0.990-0.999 correlation coefficient (R^2^) and 0.93-1.03 PCR efficiencies (E), indicating that the selected RGs in the present study for qRT-PCR are acceptable and valid for the quantification of transcripts and further experimentation (Table 1). Each RG amplified showed a high peak of a single product without any considerable nonspecific amplification in melting curve analysis. In addition, all amplicons were evaluated for primer specificity with expected product size by gel electrophoresis (Figure 2(a) and Table 1). When 10 RGs were evaluated for possible change in their mRNA levels during long-term expansion of both BM-MSCs and UCB-MSCs by qRT-PCR, a significant difference in Ct values was noted in some RGs. The HPRT1 was the only RG that showed different Ct values between E-UCB-MSCs and L-UCB-MSCs. However, many RGs including 18S, B2M, EEF1A1, GAPDH, RPLP0, and TBP showed significantly (*P *< 0.05) different Ct values during long-term expansion of BM-MSCs (Figure 2(b)). These results indicate that the expression level of RGs can be affected by long-term expansion of cells, especially in BM-MSCs.

### 3.3. Analysis of the Most Stable Reference Gene by geNorm

**Figure 3:**
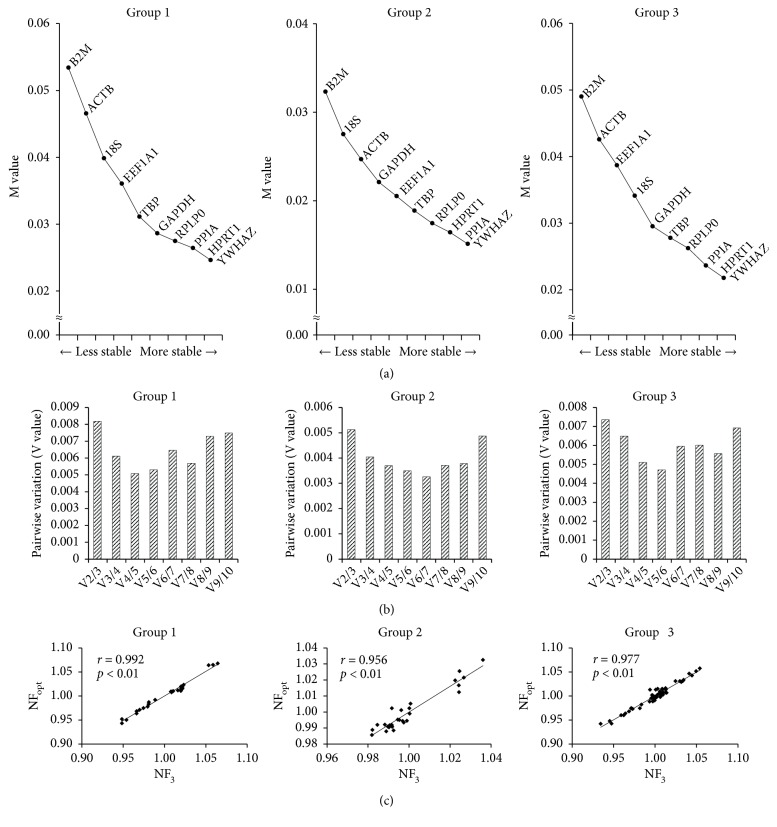
Analysis of the most stable reference gene by geNorm. (a) Stability ranking of RGs for normalization. M values were used to determine ranking of the stability of RGs where lower value indicates higher stability. (b) Optimal number of reference genes for normalization (NF_opt_). The Pairwise variation (V value) was used to determine the optimal number of RGs where lower V value indicates optimal number of RGs for normalization. The value after V on the x-axis represents the number of RGs used. (c) Correlation between NF_opt_ and NF_3_. Pearson's correlation was used to analyze correlation between NF_opt_ and NF_3_. NF_3_, the three most stable RGs for normalization. Group 1 or 2 or 3, the validation of the most stable RGs in largely expanded BM-MSCs or UCB-MSCs or BM- and UCB-MSCs, respectively.

The Ct values of long-term expanded BM-MSCs and UCB-MSCs were analyzed by geNorm to obtain M value and stable ranking of 10 RGs via gradual stepwise exclusion of the least-stable RGs. In the present study, all M values of each gene were found to be less than 1.5 indicating that all genes were reliable to use as RGs. The geNorm software suggested that YWHAZ, HPRT1, and PPIA were the 3 most stable RGs in all groups of MSCs except the traditional RGs such as GAPDH and ACTB which were found to be unstable or less stable (Figure 3(a)). Furthermore, pairwise variations by geNorm proposed that NF_opt_ in Group 1, Group 2, or Group 3 was V_4/5_, V_6/7_, and V_5/6_, respectively (Figure 3(b)). Since it is inefficient to use 4-7 RGs in the normalization of GOI, the correlation between NF_opt_ and NF_3_ was assessed to remove the excessive usage of RGs where all groups had high correlation (r > 0.9, Pearson) between NF_3_ and NF_opt_ (Figure 3(c)).

### 3.4. Analysis of the Most Stable Reference Gene by NormFinder

**Figure 4:**
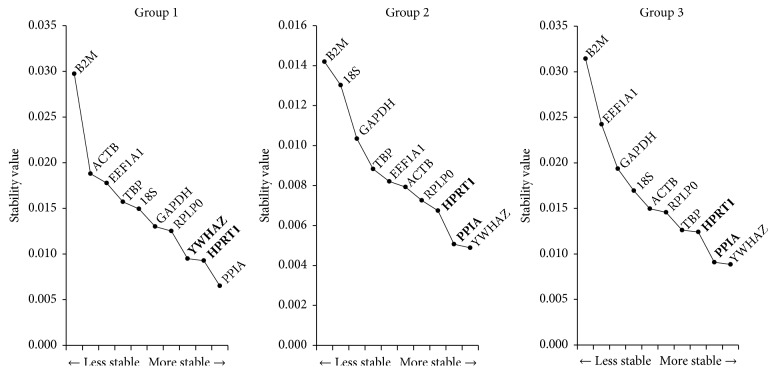
Analysis of the most stable reference gene by NormFinder. The stability values of 10 selected RGs in long-term expanded BM-MSCs and UCB-MSCs were calculated by NormFinder. The y-axis represents the stability and the lower values indicates the higher stability. The best combinations of two RGs in each group were determined by NormFinder, and are marked with bold letters.

Using NormFinder analysis program, the PPIA or YWHAZ were determined as the most stable reference genes in Group 1 or Group 2 and 3, respectively. Furthermore, when intergroup variations were considered, the best combination of two genes from the pool of 10 RGs was YWHAZ with HPRT, or PPIA with HPRT in Group 1 or Group 2 and 3, respectively (Figure 4). In consistent with the results obtained from geNorm, NormFinder showed that YWHAZ, HPRT1, and PPIA were the most stable RGs during long-term expansion of BM-MSCs and UCB-MSCs while traditional RGs were less stably expressed. A slight difference obtained in the rankings of 10 RGs between geNorm and NormFinder may possibly due to the different algorithms used.

### 3.5. Use of Most Stable Reference Genes for Normalization of GOI

**Figure 5:**
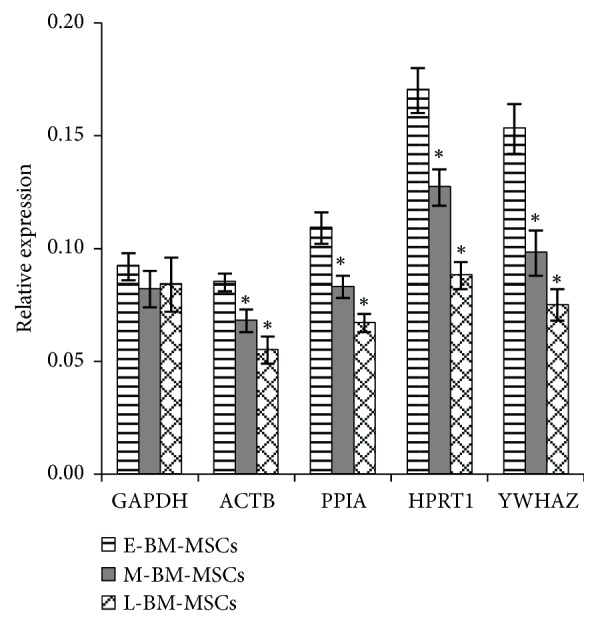
Application for normalization of the most stable reference genes. OCT4 gene expression level in long-term expanded BM-MSCs was normalized using most stable RGs (PPIA, HPRT1 and YWHAZ) validated by geNorm and NormFinder, and two traditional RGs (GAPDH and ACTB) to demonstrate the effect of validated RGs on normalization of gene of interest. The data were presented as mean ± SD. *∗P* < 0.05 vs E-BM-MSCs.

Based on the results obtained in Figures 1 and 2, it is likely that long-term expansion under defined culture conditions has affected the characteristics of BM-MSCs in terms of their differentiation and proliferative ability, degree of cellular senescence, and RG expression levels. Therefore, to verify the influence of RGs on normalization of gene of interest, the expression of OCT4, a key pluripotency-related gene, was normalized against the three most stable RGs (PPIA, HPRT1, and YWHAZ) determined in the present study and the less stable traditional RGs (ACTB and GAPDH) in E-BM-MSCs, M-BM-MSCs, and L-BM-MSCs. As a result, OCT4 expression in L-BM-MSCs was significantly (*P *< 0.05) decreased when compare to that in E-BM-MSCs when PPIA, HPRT1, YWHAZ, and ACTB were used for normalization. However, the traditional RG GAPDH showed no significant difference in OCT4 expression between E-BM-MSCs, M-BM-MSCs, and L-BM-MSCs (Figure 5).

## 4. Discussion

As MSCs are currently promising regimens for regenerative medicine and immunomodulatory agents, the use of suitable RGs is indispensable to assess the accurate and reliable gene expression data [10, 11, 21]. In addition, since long-term expansion of MSCs under defined culture conditions and the effect of such* in vitro* environments on cells must be studied prior to their application. Hence, the expression stability of 10 commonly used RGs was evaluated in long-term expanded BM-MSCs and UCB-MSCs under defined culture conditions through widely used RG validation programs (geNorm and NormFinder). Both programs suggested that PPIA, HPRT1, and YWHAZ are the three most stable RGs regardless of* in vitro* culture expansion period.

The long-term expanded MSCs display limited proliferation ability similar to somatic cells may be due to the replicative senescence [25–27]. The senescent MSCs display not only altered proliferation ability but also morphological changes, loss of differentiation capacity, increased expression of SA-*β*-gal, elevated oxidative stress, epigenetic modifications, telomere shortening, DNA damage, and change in genes expression profile [27–32]. In the present study, BM-MSCs showed enlarged and irregular morphologies, gradual loss in trilineage differentiation capacity and proliferative ability, and increased senescence-associated SA-*β*-gal marker expression, while UCB-MSCs maintained their phenotypes through early passages to late passages (Figure 1). Although, the decreased expression of CD105 during long-term expansion of BM-MSCs was not significant, this slight decrease in the expression may be due to the unknown consequences under serum free culture conditions [33]. In addition, the expression of several RGs in BM-MSCs was affected by long-term expansion. However, the HPRT1 was the only gene affected in the long-term expanded UCB-MSCs under present experimental conditions. Since HPRT1 known to play a central role in purine salvage pathway, cell cycle, and proliferation mechanisms, its change in expression in L-UCB-MSCs may possibly be due to a compensatory mechanism to maintain proliferative ability under defined culture conditions (Figures 1(d) and 2(b)) [34, 35]. Overall, the long-term expansion of MSCs resulted in the alteration of cellular characteristics as well as the expression of RGs.

The validation of most stable RGs under each experimental condition is an essential step to obtain accurate and reliable results by qRT-PCR because there is no single RG that is universally stable in its expression under all defined culture conditions. The present study also demonstrated that the expression level of RGs could be affected by experimental conditions as evident in Figure 2(b). For instance, although ACTB and GAPDH are the basic cell survival factors, their expression level was found to change as high as seventeen times based on culture conditions and culture duration [5, 6, 20, 36]. Therefore, the selection of nonvalidated or unstable RGs during normalization of GOI may lead to inconsistent or misleading results [11, 37]. The incorrect selection of RGs drastically affects the results [4, 11, 22, 38]. When the expression of lineage-specific markers in differentiated MSCs toward adipocytes, osteoblasts, and chondrocytes was normalized against the most stable RGs (RPLP0 and EEF1A1 for osteogenesis; TBP and YWHAZ for adipogenesis and chondrogenesis) and the least stable RG (EEF1A1 for adipogenesis; B2M for osteogenesis and chondrogenesis), inconsistent results of the relative expression of lineage-specific markers were significantly (*p *< 0.0001) identified. Especially, normalization against the least stable RGs in differentiated MSCs toward osteoblasts and chondrocytes resulted in less fold change in the expression compared to that when using the most stable RGs [4].. In addition, the relative gene expression levels of chondrogenic markers or OCT4 from chondrogenesis-induced ATDC5 cells (progenitor cells for chondroblasts) or different types of porcine blastocysts were found to be varied depending on the use of RGs, respectively [22, 38]. The present study also demonstrated an inconsistence in results obtained between stable RGs (PPIA, HPRT1 and YWHAZ) and the traditional RGs (ACTB and GAPDH) during long-term expansion of MSCs under defined culture conditions. Considering the occurrence of loss of stemness during long-term expansion of BM-MSCs, the expression of OCT4 in L-BM-MSCs was reduced when compared to E-BM-MSCs when three most stable RGs verified in the present study were used; however, no significant difference in the expression of OCT4 was noted in BM-MSCs irrespective of culture period when GAPDH was used for normalization (Figure 5). Therefore, these results indicate that the validation and use of stable RGs under each experimental condition are critical for accurate analysis of qRT-PCR in order to avoid the false conclusions resulting from the use of invalidated RGs.

The validation of Ct values obtained by qRT-PCR using well-established algorithms such as geNorm, NormFinder, and BestKeeper is necessary to investigate the stability of RGs. Recently, several studies have evaluated the most stable RGs in human MSCs or progenitor cells [4–6, 20, 39, 40]. The importin 8 (IPO8), cancer susceptibility candidate 3 (CASC3), and TBP were recommended as RGs for studies on osteoarthritis patient-derived BM-MSCs [39]. Further, it has been demonstrated that the expression of TBP and YWHAZ is found to be stable in BM-MSCs and adipose tissue-derived MSCs (AT-MSCs) regardless of treatments such as supplementation of vascular endothelial growth factor (VEGF) to potentially enhance the properties of MSCs [40]. Considering that the studies involving comparative characterization of various source derived MSCs including an evaluation of stable RGs under varied differentiation status are prerequisite to define the most appropriate cell source for clinical applications. The expression of RPL13A in AT-MSCs and Wharton's jelly-derived MSCs (WJ-MSCs) and HPRT1 in BM-MSCs has been validated as stable and the expression of TBP, YWHAZ, glucuronidase beta (GUSB), and RPL subtypes has been identified as most stable for UCB-MSCs, BM-MSCs, and AT-MSCs with or without induction of differentiation [4, 5]. Further, PPIA, B2M and RPL13A are recommended to use as RGs for BM-MSCs and fetal tissue-derived MSCs (FT-MSCs) [6]. In general, RPL subtypes are found to be more stable with minor inconsistency among donors when compared to other RGs [20]. The different experimental conditions such as cell source, donor, treatment, differentiation induction, and cell expansion may have profound effect on the stability of RGs. Therefore, it is important to verify the most stable RGs under each experimental condition before using for further studies. The present study recommends the use of PPIA, HPRT1, and YWHAZ for normalization of GOI in qRT-PCR experiments to get accurate and reliable results as determined by geNorm and NormFinder analysis for long-term expanded BM-MSCs and UCB-MSCs [Figures 3 and 4].

Previously, several RGs such as ACTB and GAPDH were traditionally used for normalization as they were thought to be constantly expressed in different cell lines and conditions [5]. However, these traditional RGs have been reported to be unstable in many studies with MSCs [4, 5, 20, 39, 41]. The analysis of higher maximum fold change (MFC) of ACTB among other RGs in osteogenesis-induced BM-MSCs indicated the expression of ACTB was associated with morphological changes of differentiated MSCs and found to be unstable during osteogenesis [41]. In addition, the lower stability of ACTB among pool of candidate RGs was demonstrated in AT-MSCs, BM-MSCs, WJ-MSCs, and UCB-MSCs under different experimental conditions [4, 5, 20, 39]. Similar to ACTB, the expression of another RG GAPDH was also found to be variable or less stable in AT-MSCs and BM-MSCs [5, 42, 43]. In consistency with these earlier findings, the present study also demonstrated that ACTB and GAPDH were less stable in long-term expanded BM-MSCs and UCB-MSCs and were not always successful in all normalization data. Both geNorm and NormFinder analysis found that the stability rankings of ACTB and GAPDH were placed in fifth to ninth among the pool of 10 RGs. In addition, GAPDH led to inconsistent or misleading results during normalization of OCT4 [Figure 5]. These results indicated that both ACTB and GAPDH are not suitable RGs with regard to gene expression analysis in long-term expanded BM-MSCs and UCB-MSCs under defined culture conditions and, therefore, highlighting the importance of validation of RGs.

In conclusion, the present study demonstrated that the three RGs, PPIA, HPRT1, and YWHAZ among the pool of 10 RGs are more suitable for normalizing the expression of GOI and that the traditional RGs such as ACTB and GAPDH are identified as less stable under current experimental conditions. To our knowledge, this is the first kind of its study to evaluate the stability of the expression of RGs under multifactorial conditions with respect to cell source, long-term expansion until passage 20, and defined media culture conditions.

## Figures and Tables

**Table 1:** Information of primers of candidate reference genes and their standard curve parameters.

Information of primers				Standard curve parameters			
Gene name (Symbol)	Sequence	Pro. size	Reference	R^2^	M	B	E
18S ribosomal RNA (18S)	F: CACGGACAGGATTGACAGATTG R: AACCAGACAAATCGCTCCAC	92	X03205.1	0.998	-3.507	34.251	0.95

Hypoxanthine phosphoribosyltransferase 1 (HPRT1)	F: CGAGATGTGATGAAGGAGATGG R: TGATGTAATCCAGCAGGTCAGC	132	NM_000194.2	0.999	-3.499	32.221	1.01

Beta-2-microglobulin (B2M)	F: CAGCGTACTCCAAAGATTCAGG R: GGATGAAACCCAGACACATAGC	116	NM_003406.3	0.995	-3.452	37.651	1.01

Eukaryotic translation elongation factor 1 alpha 1 (EEF1A1)	F: ACTATCATTGATGCCCCAGGAC R: ACACCAGCAGCAACAATCAG	121	NM_001402.5	0.991	-3.523	35.587	0.97

Ribosomal protein, large, P0 (RPLP0)	F: TGGGCAAGAACACCATGATG R: CGGATATGAGGCAGCAGTTTC	98	NM_001002.3	0.998	-3.511	37.016	0.96

Peptidylprolyl isomerase A (PPIA)	F: TGCTGGACCCAACACAAATG R: AACACCACATGCTTGCCATC	89	NM_021130.3	0.990	-3.388	33.650	1.03

Actin. Beta (ACTB)	F: ACACCAGCAGCAACAATCAG R: CGGATATGAGGCAGCAGTTTC	126	NM_001002.3	0.997	-3.447	36.891	0.99

Tyrosine 3-monooxygenase/tryptophan 5- monooxygenase activation protein, zeta (YWHAZ)	F: CGAAGCTGAAGCAGGAGAAG R: TTTGTGGGACAGCATGGATG	111	NM_003406.3	0.995	-3.356	34.613	0.94

Glyceraldehyde-3-phosphate dehydrogenase (GAPDH)	F: ACGAAGCTGAAGCAGGAGAAG R: GGATGAAACCCAGACACATAGC	105	NM_002046.4	0.996	-3.486	39.012	0.93

TATA box binding protein (TBP)	F: AACCAGACAAATCGCTCCAC R: GGATGGGCATCATGGAAAC	114	NM_003406.3	0.996	-3.374	33.282	1.03

Octamer-binding transcription factor 4 (OCT4)	F: ACATCAAAGCTCTGCAGAAAGA R: AATACCTTCCCAAATAGAACCC	127	NM_002701	-	-	-	-

Pro. Size, product size (base pair); R2, correlation; M, slope; B, intercept; E, efficiency

## Data Availability

All the data related to the current manuscript can be made available upon request whenever needed.

## Supplementary Materials

Supplementary 1 Supplementary Figure 1: Cell surface marker analysis of MSC-positive markers (CD44, CD105, and Vimentin) and negative markers (CD34 and CD45) in long-term expanded BM-MSCs. Here E-BM, M-BM, and L-BM represent the early, middle, and late bone marrow MSCs.

Supplementary 2 Supplementary Figure 2: Cell surface marker analysis of MSC-positive markers (CD44, CD105, and Vimentin) and negative markers (CD34 and CD45) in long-term expanded UCB-MSCs. Here E-UCB, M-UCB, and L-UCB represent the early, middle, and late umbilical cord blood MSCs.
