# Supplementary figures and images for: PPIA, HPRT1, and YWHAZ Genes Are Suitable for Normalization of mRNA Expression in Long-Term Expanded Human Mesenchymal Stem Cells
Source: Biomed Res Int. 2019 May 21;2019:3093545. doi: 10.1155/2019/3093545 (PMC6556274; doi:10.1155/2019/3093545)

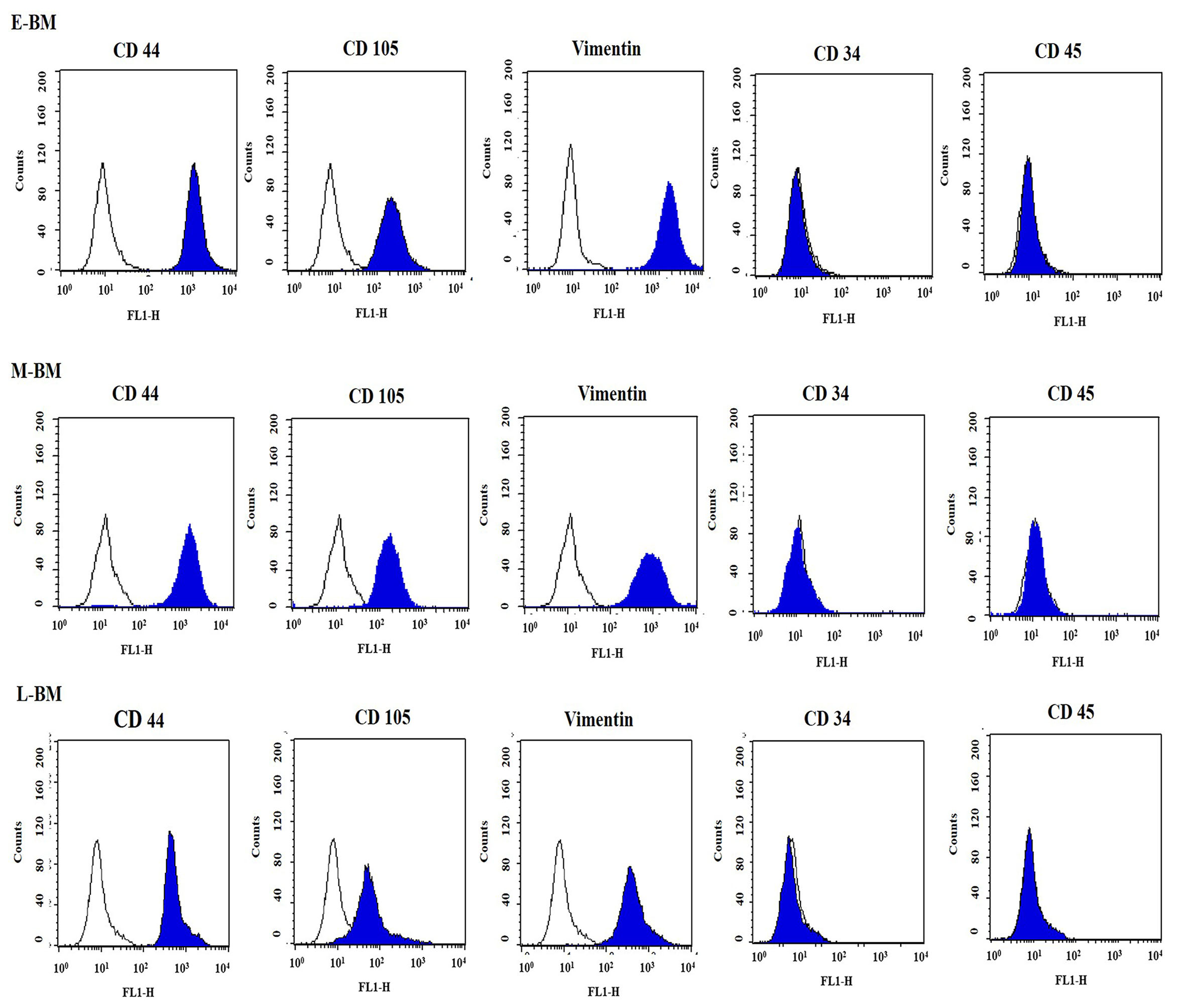

Supplement: Supplementary 1 — Supplementary Figure 1: Cell surface marker analysis of MSC-positive markers (CD44, CD105, and Vimentin) and negative markers (CD34 and CD45) in long-term expanded BM-MSCs. Here E-BM, M-BM, and L-BM represent the early, middle, and late bone marrow MSCs. [file 3093545.f1.jpg]

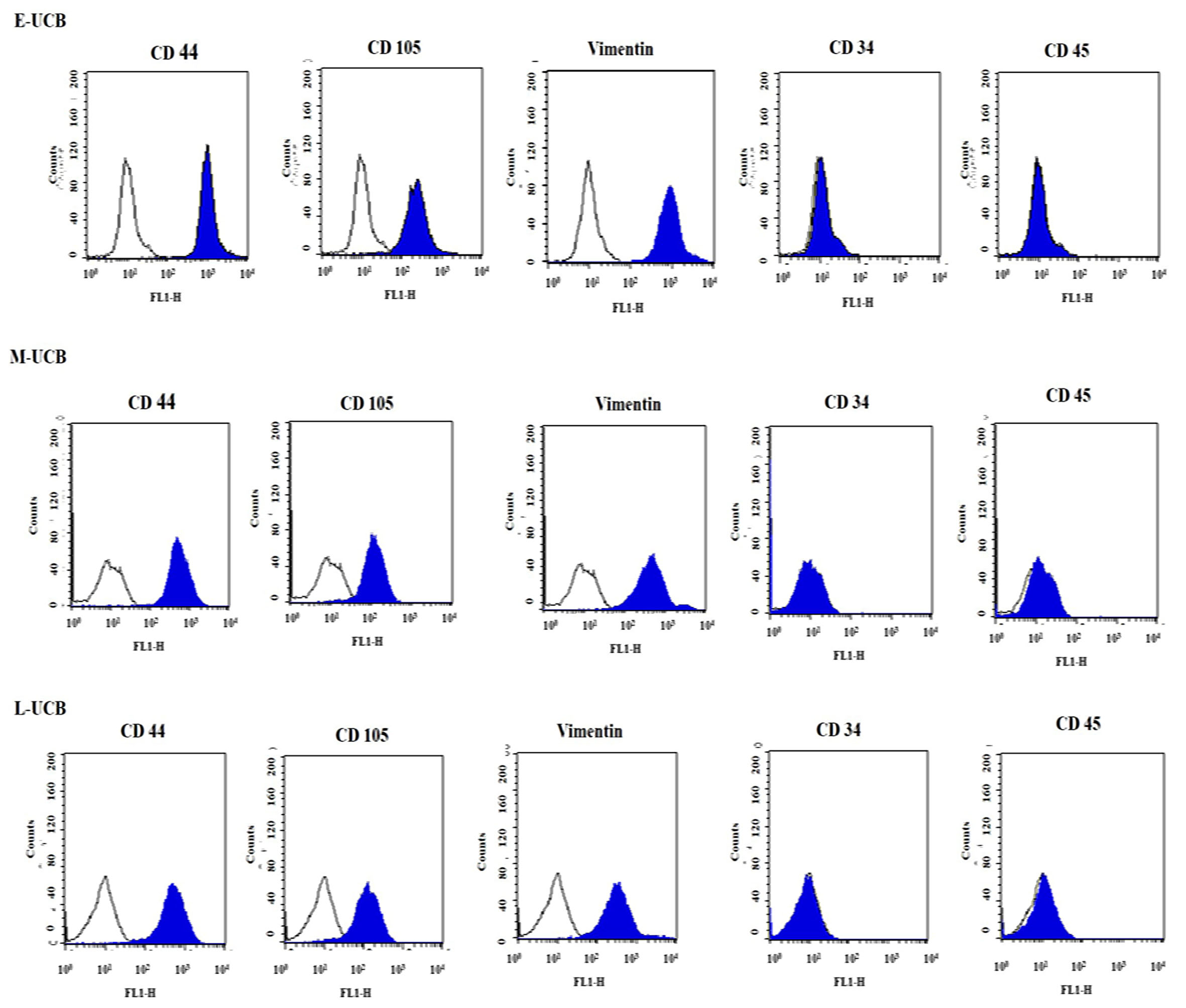

Supplement: Supplementary 2 — Supplementary Figure 2: Cell surface marker analysis of MSC-positive markers (CD44, CD105, and Vimentin) and negative markers (CD34 and CD45) in long-term expanded UCB-MSCs. Here E-UCB, M-UCB, and L-UCB represent the early, middle, and late umbilical cord blood MSCs. [file 3093545.f2.jpg]
